# Antibiotic pretreatment with ampicillin or streptomycin differentially shapes *Salmonella* infection kinetics and associated intestinal inflammation in mice

**DOI:** 10.1080/19490976.2026.2725469

**Published:** 2026-09-03

**Authors:** Luca Maurer, Ursina Enz, Ersin Gül, Patrick Kiefer, Philipp Christen, Lea Fuchs, Wolf-Dietrich Hardt

**Affiliations:** a Institute of Microbiology, Department of Biology, ETH Zürich, Zürich, Switzerland

**Keywords:** *Salmonella* Typhimurium, enteric infections, mouse model, gut microbiota, antibiotic pretreatment, streptomycin, ampicillin, intestinal inflammation

## Abstract

*Salmonella* *enterica* serovar Typhimurium (*S*. Tm) mouse infection models commonly rely on antibiotic pretreatment to disrupt microbiota-mediated colonization resistance and enable reproducible infection. Streptomycin is the primary antibiotic used, but alternative pretreatment regimens like ampicillin are increasingly employed, particularly for infections with attenuated *S*. Tm mutants. However, the impact of different antibiotic pretreatments on *Salmonella* infection kinetics and host responses remains poorly characterized. Here, we compared *S*. Tm infection outcomes in streptomycin- versus ampicillin-pretreated mice. While both antibiotics supported comparable luminal colonization, ampicillin pretreatment induced earlier, stronger, and more sustained intestinal inflammation and histopathology during *S*. Tm infection. In germ-free mice, these differences were absent, indicating a microbiota-dependent mechanism, further supported by microbiota transfer experiments. The distinct infection outcomes were associated with antibiotic-specific alterations in the microbiota composition, while targeted analysis of luminal metabolites by mass spectrometry revealed no pronounced differences. Strikingly, ampicillin pretreatment coincided with higher early TTSS-1 expression in *S*. Tm, consistent with increased epithelial invasion. Together, our findings demonstrate that antibiotic pretreatment profoundly shapes the intestinal environment and is associated with differences in *Salmonella* virulence expression, host responses, and infection kinetics. The choice of antibiotic pretreatment is therefore a crucial variable in murine *Salmonella* infection models.

## Introduction

*Salmonella enterica* serovar Typhimurium (*S.* Tm) is a tractable and widely used model organism for studying aspects of enteric pathogenesis, such as bacterial invasion strategies, gut inflammation, and host defense mechanisms. Successful infection relies on the coordinated action of two type III secretion systems (TTSS) encoded on *Salmonella* pathogenicity islands (SPI). TTSS-1 mediates invasion of intestinal epithelial cells,^1-3^ while TTSS-2 supports intracellular survival and replication within host cells.^4-9^

Although only a small fraction of luminal *S*. Tm actively invades the epithelium,^10^ this is sufficient to trigger a robust inflammatory response driven by SPI-1- and SPI-2-dependent mechanisms.^8^ ^,^ ^11^ Inflammation induces the production of antimicrobial effectors, such as reactive oxygen and nitrogen species, disrupting both the pathogen and commensal microbiota members.^12-14^ Consequently, this broad antimicrobial activity further disrupts the microbial community, weakening colonization resistance, the ability of the resident microbiota to prevent pathogen establishment.^15^ At the same time, inflammation increases the availability of oxygen and alternative respiratory electron acceptors, thereby creating a niche that favors the expansion of luminal *S.* Tm populations.^15^

In experimental mouse models, microbiota differences between individuals lead to variable susceptibility to *S*. Tm colonization. To bypass colonization resistance and ensure reproducible infection outcomes, mice are commonly pretreated with high doses of antibiotics prior to oral challenge.^16^ The streptomycin-pretreated mouse model is the most widely used approach, as streptomycin selectively depletes obligate anaerobic commensals and transiently promotes the expansion of facultative anaerobes such as Enterobacteriaceae, creating a permissive environment for *S*. Tm colonization and inflammation.^17-19^ As a result, the streptomycin model has become a cornerstone of experimental *Salmonella* research over the past two decades.

However, in experiments with attenuated *S.* Tm mutants, streptomycin may, in some cases, only incompletely alleviate colonization resistance for reasons that remain unclear. If unrecognized, this may lead to mechanistic misinterpretation of the observed phenotypes. In such situations, alternative regimens, such as ampicillin pretreatment, have been employed to assess infection kinetics, particularly in studies involving SPI-2–deficient strains.^20-28^ SPI-2-deficient *S.* Tm (*S*. Tm^SPI2^) are commonly used strains in addition to wild-type *S*. Tm (*S*. Tm^WT^) in *Salmonella* research. While both wild-type and SPI-2-deficient *S*. Tm invade the gut tissue and promote similar inflammation levels during early infection, the absence of SPI-2 attenuates disease progression in later stages of infection.^8^ ^,^ ^24^

Ampicillin, a broad-spectrum *β*-lactam antibiotic, targets both Gram-positive and some Gram-negative bacteria, leading to a profound reduction in microbial diversity and compositional shifts.^29^ ^,^ ^30^ Despite the widespread use of ampicillin pretreatment, the functional consequences of different antibiotic pretreatments on the intestinal environment and subsequent *S*. Tm infection dynamics have not been systematically defined.

Given that results obtained under different pretreatment regimens are often interpreted within the same conceptual framework, it is essential to determine whether they are functionally equivalent or fundamentally distinct. Here, we compared the outcomes of *Salmonella* infection following ampicillin or streptomycin pretreatment across multiple mouse lines. We show that antibiotic-dependent alterations of the gut ecosystem influence host inflammation and *S.* Tm virulence programs.

## Results

### Ampicillin pretreatment results in stronger intestinal inflammation than streptomycin pretreatment during *S*. Tm^SPI2^ infection across two host genotypes and backgrounds

**Figure 1.**
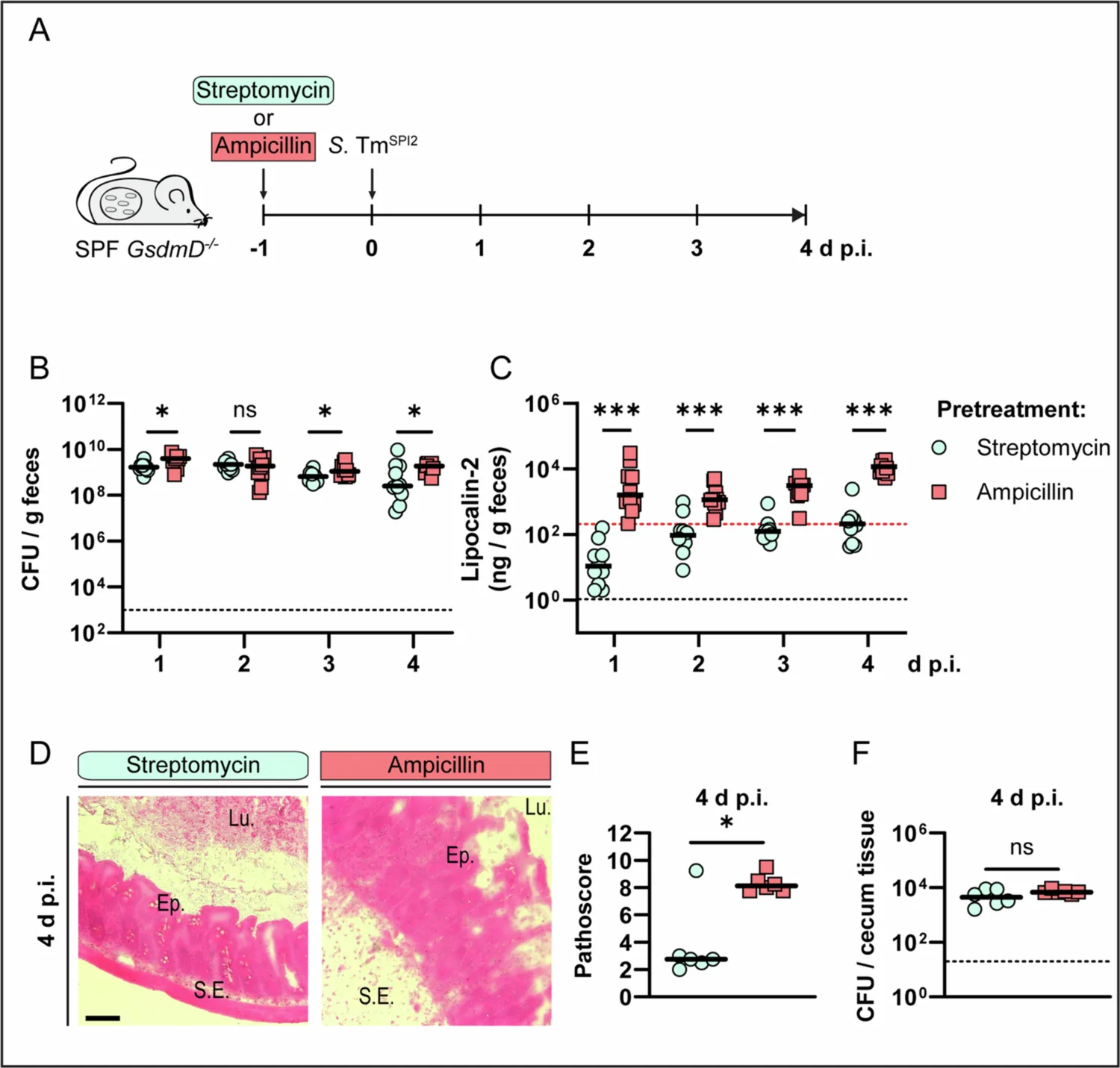
Ampicillin pretreatment promotes pronounced inflammation during *S*. Tm^SPI2^ infection. 4 d *S*. Tm^SPI2^ infection of *GsdmD* ^−/−^ mice (C57BL/6 background) pretreated with either streptomycin (green circles) or ampicillin (red squares). (A) Experimental setup. (B) *S*. Tm^SPI2^ pathogen loads in feces. (C) Quantification of inflammation by LCN2 levels of feces. The red dotted line indicates 200 ng/g feces, the threshold used to define inflammation. (D) Representative micrographs of H&E-stained cecal tissue sections at 4 d p.i. Ep. epithelium, Lu. lumen, S.E. submucosa edema. Scale bar: 100 μm. (E) Microscopy-based quantification of histology scores from H&E-stained cecum tissue sections. (F) *S*. Tm^SPI2^ pathogen loads in cecum tissue. In B, C, E, and F, each data point represents one mouse (B, C: *n* = 10 per group, pooled from 2 independent experiments; E, F: *n* = 6 per group, subset of B, C). Line at the median. The dotted line represents the detection limit. Mann‒Whitney U test (ns – not significant, *P < 0.05, ***P < 0.001).

To assess whether streptomycin and ampicillin pretreatment differentially affect *S.* Tm infection dynamics, we compared pathogen colonization and host pathology following these two commonly used antibiotic pretreatment regimens. A previous study suggested that different mouse strains exhibit distinct responses to antibiotic perturbation and subsequent infection, likely due to differences in gut microbiota composition or host genetic background, although the underlying cause could not be fully resolved.^31^ Given this, we included wild-type (WT) and Gasdermin D-deficient (*GsdmD* ^*−/−*^) mice; in the latter, we had previously observed that infection with attenuated *S.* Tm after streptomycin pretreatment does not lead to pronounced intestinal inflammation. Accordingly, both WT and *GsdmD* ^*−/−*^ mice were pretreated with a single dose of either streptomycin or ampicillin. One day later, the mice were infected orally with 5 × 10^7^ colony-forming units (CFU) *S*. Tm^SPI2^ for 4 days (Figures 1A and S1A). In WT C57BL/6 mice, both pretreatments supported efficient luminal colonization (Figure S1B). Streptomycin pretreatment resulted in a slight decline in fecal pathogen loads in some mice by day 4 p.i. In contrast, the ampicillin-pretreated mice maintained more stable, full colonization, defined as retaining fecal loads above the 10^9^ CFU/g threshold throughout the experiment, resulting in less inter-individual biological variation within this group (Figure S1B). Moreover, fecal lipocalin-2 (LCN2) levels, a well-established marker of intestinal inflammation, were significantly elevated in ampicillin-pretreated mice compared to streptomycin-pretreated mice. By day 1 p.i., ampicillin-pretreated mice reached ~10^4^ ng LCN2/g feces, while LCN2 levels of most streptomycin-pretreated WT mice remained below 100 ng/g and only reached ~10^3^ ng/g by day 4 (Figure S1C). In *GsdmD* ^*−/−*^ mice, similar trends were observed for luminal colonization. Ampicillin-pretreated *GsdmD* ^*−/−*^ mice maintained higher fecal *S*. Tm^SPI2^ counts (~10^9^ CFU/g) at days 1, 3, and 4 p.i. than streptomycin-pretreated mice, in which some animals showed a steep decline to below 10^8^ CFU/g by day 4 p.i. (Figure 1B). Furthermore, the difference in LCN2 levels between pretreatments was far more pronounced in *GsdmD* ^*−/−*^ mice than in WT mice: LCN2 levels in the ampicillin group remained stable at ~10^4^ ng/g feces throughout the course of infection (Figure 1C). In contrast, fecal LCN2 levels in streptomycin-pretreated *GsdmD* ^*−/−*^mice gradually increased over time, reaching ~200 ng/g at day 4 p.i., which was approximately fivefold lower than the levels observed in streptomycin-pretreated WT mice infected with *S*. Tm^SPI2^ (compare Figures 1C and S1C). Consistent with the LCN2 data, histopathological analysis of *GsdmD* ^*−/−*^ mice revealed significantly higher scores in ampicillin-pretreated mice (Figure 1D,E), despite similar *S*. Tm^SPI2^ CFU loads in cecum tissue at day 4 p.i. (Figure 1F).

Taken together, these data show that, in contrast to streptomycin pretreatment, ampicillin pretreatment in combination with *S*. Tm^SPI2^ infection induces a robust and sustained intestinal inflammatory response in mice. Notably, this phenotype was observed in both WT and *GsdmD* ^*−/−*^ mice, indicating that it is not restricted to a specific host genotype. Importantly, the broader generalizability of this antibiotic-dependent difference in *Salmonella*-induced gut inflammation was further confirmed in an alternative host background using 129S6/SvEvTac mice infected with *S*. Tm^SPI2^ (Figure S1D–F). While the phenotype was robust across host backgrounds, the effect was more pronounced in *GsdmD* ^*−/−*^ mice than in WT C57BL/6 mice, indicating a difference in the magnitude of the response between the two genotypes. These varying magnitudes of inflammation between our C57BL/6 genotypes raised the question of whether the phenotype is primarily driven by the host genotype or rather by microbiota-dependent effects. Since addressing this question could help to better understand the mechanisms underlying the differential infection outcomes, we focused our subsequent analyses on *GsdmD* ^*−/−*^ mice.

### Antibiotic-dependent *S*. Tm^SPI2^ infection phenotypes are independent of host GSDMD status

**Figure 2.**
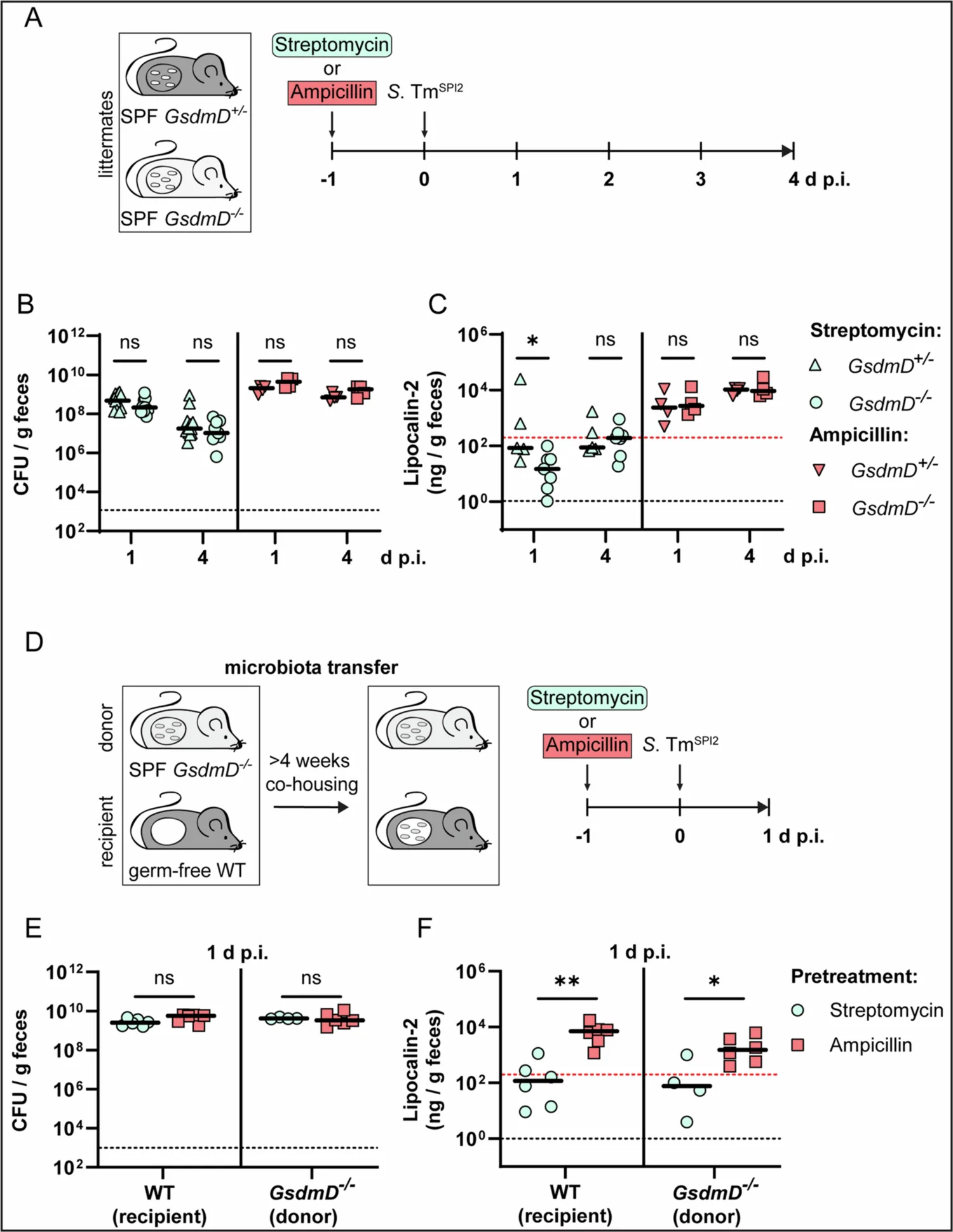
*S*. Tm ^SPI2^ infection phenotypes are antibiotic-dependent but GSDMD-independent. (A−C) 4 d *S*. Tm^SPI2^ infection of littermate *GsdmD* ^*+/−*^ and *GsdmD* ^−/−^ mice (C57BL/6 background) pretreated with either streptomycin or ampicillin. Streptomycin-pretreated mice are indicated in green (*GsdmD* ^*+/−*^, triangles; *GsdmD* ^−/−^, circles), and ampicillin-pretreated mice are indicated in red (*GsdmD* ^*+/−*^, inverted triangles; *GsdmD* ^−/−^, squares). (A) Experimental setup. (B) *S*. Tm^SPI2^ pathogen loads in feces at 1 d p.i. and 4 d p.i. (C) Quantification of inflammation by LCN2 levels of feces. The red dotted line indicates 200 ng/g feces, the threshold used to define inflammation. (D–F) Germ-free (GF) WT mice (recipient) were co-housed with SPF *GsdmD* ^−/−^ mice (donor) to allow microbiota transfer (>4 weeks) and subjected to streptomycin or ampicillin pretreatment prior to *S*. Tm^SPI2^ infection for 1 d. (D) Experimental setup. (E) *S*. Tm^SPI2^ pathogen loads in feces at 1 d p.i. of recipient (left) and donor (right) mice. (F) Quantification of inflammation by LCN2 levels of feces. The red dotted line indicates 200 ng/g feces, the threshold used to define inflammation. In B, C, E, F, each data point represents one mouse (B, C: streptomycin *GsdmD* ^*+/−*^ *n* = 12, *GsdmD* ^−/−^ *n* = 9, ampicillin *n* = 4 each; E, F: WT (recipient) *n* = 6 each, *GsdmD* ^−/−^ (donor) streptomycin *n* = 4, ampicillin *n* = 6). Data from  ≥ 2 independent experiments were used for each comparison. In A–C, data for streptomycin and ampicillin treatments are derived from independent experimental cohorts. Line at the median. The dotted line represents the detection limit. Mann-Whitney U test (ns – not significant, *P < 0.05).

To test whether the observed inflammation and colonization phenotypes are influenced by the host genotype (GSDMD-deficiency), we followed two different approaches. First, we repeated the experiment in *GsdmD* ^*−/−*^mice and their heterozygous littermates (*GsdmD* ^*+/−*^) (Figure 2A), which express GSDMD and are thus widely used as a proxy for WT mice in infection studies.^32^ Overall, GSDMD-deficiency did not substantially alter the antibiotic-driven differences in inflammation or fecal pathogen loads (Figure 2B,C). Ampicillin-pretreated animals consistently exhibited stable fecal pathogen loads of ~10^9^ CFU/g and increasing inflammation levels of up to 10^4^ ng LCN2/g feces over the course of infection. In contrast, the streptomycin-pretreated animals showed lower fecal pathogen loads at day 1 p.i. that further declined to ~10^7^ CFU/g at day 4 p.i. while inflammation levels remained low throughout the infection (~200 ng LCN2/g feces). Notably, at day 1 p.i., streptomycin-pretreated *GsdmD* ^*+/−*^ mice displayed modestly higher LCN2 levels compared to *GsdmD* ^*−/−*^ mice, consistent with the results presented in Figures 1 and S1, as well as with previous observations that GSDMD promotes early gut inflammation during *S*. Tm^WT^ infection.^32^

Second, we co-housed germ-free (GF) WT mice (recipient) with *GsdmD* ^*−/−*^ mice (donor) for >4 weeks to allow microbiota transfer, a well-established method for the horizontal transfer of microbes, though it does not necessarily reproduce the donor's exact microbial composition.^33-36^ While the detailed taxonomic composition and exact transfer efficiency of individual microbial taxa were not analyzed, successful microbial colonization was confirmed via qPCR analysis of total fecal 16S rRNA gene levels (Figure S2A). Thereafter, these mice were subjected to streptomycin or ampicillin pretreatment prior to 1 d of *S*. Tm^SPI2^ infection (Figure 2D). The WT mice (= recipients), having acquired their microbiota from the *GsdmD* ^*−/−*^ donor colony via co-housing, exhibited antibiotic-dependent phenotypes comparable to those observed in *GsdmD* ^*−/−*^ mice (donor) (Figure 2E,F). This experiment demonstrated that GSDMD-deficiency is not required for the observed phenotypes and instead supports a role for microbiota-dependent changes in response to the antibiotic treatments.

In summary, these results suggest that although GSDMD can modestly affect early inflammatory responses in certain contexts, pronounced differences in *S*. Tm^SPI2^-induced gut inflammation between streptomycin and ampicillin pretreatment are largely independent of host genotype (i.e., GSDMD-deficiency) and may instead reflect antibiotic-specific alterations of the gut microbiota or other microbiota-associated effects. Therefore, the more pronounced effect in *GsdmD* ^*−/−*^ mice compared to WT C57BL/6 mice likely reflects colony-specific differences in microbiota composition.

### Distinct microbiota profiles emerge following ampicillin or streptomycin pretreatment

Earlier work had shown that different antibiotics can affect the gut microbiota-conferred colonization resistance to *Salmonella* infections in different ways.^37^ Based on these findings, we hypothesized that differences in disease progression between streptomycin- and ampicillin-pretreated mice are attributable to antibiotic-specific alterations to the gut microbiota. To test whether the gut microbiota is required for this phenotype, we pretreated GF mice with either streptomycin or ampicillin prior to *S*. Tm^SPI2^ infection for 1 d (Figure S3A). Strikingly, we did not observe any significant difference between streptomycin- and ampicillin-pretreated GF animals with respect to luminal colonization and LCN2 levels (Figure S3B,C). Complementing our co-housing data (Figure 2E,F), these data confirm that the observed differences between streptomycin- and ampicillin-pretreated mice (Figures 1 and S1) are microbiota-dependent and not driven by direct antibiotic effects in the absence of a microbial community.

**Figure 3.**
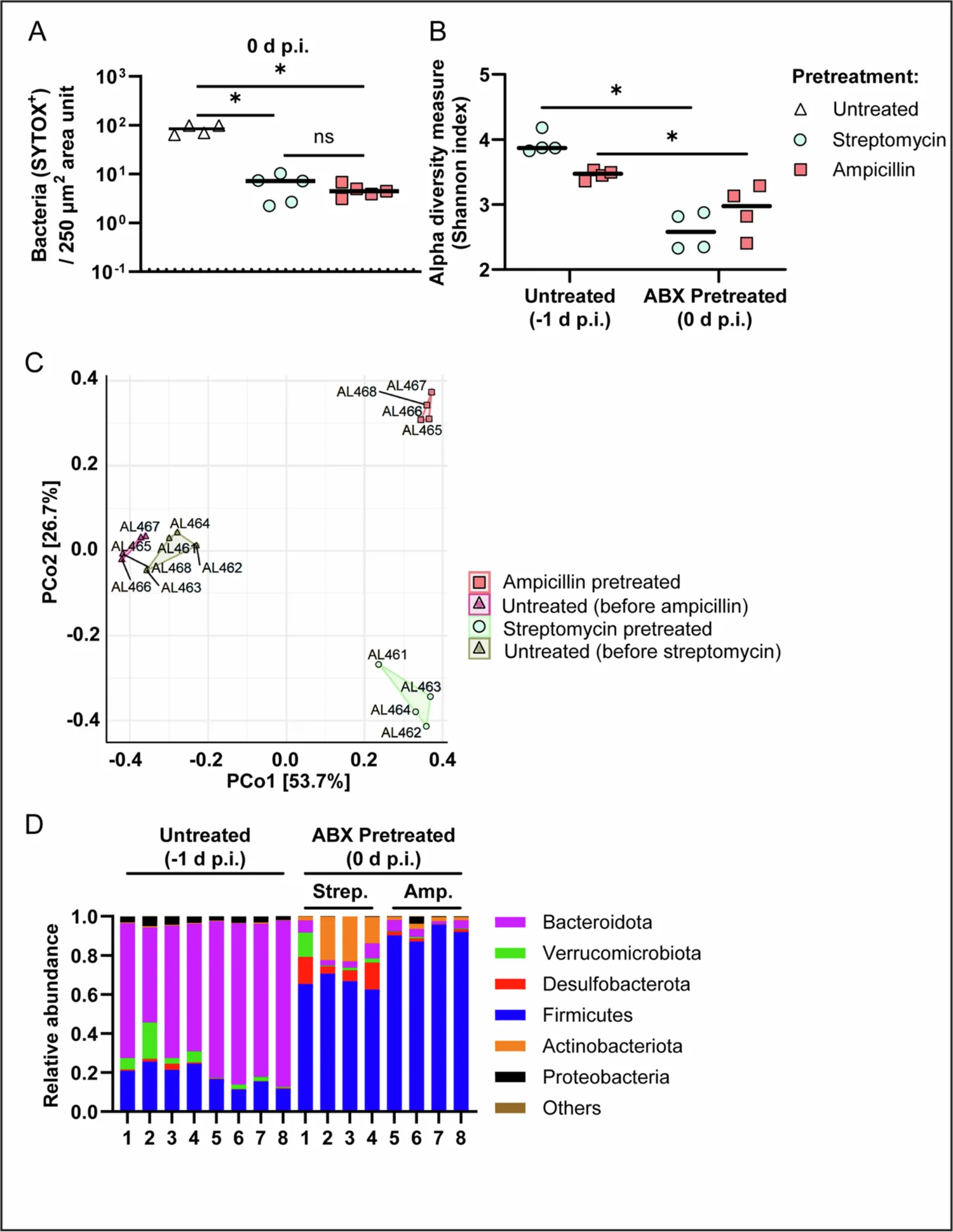
Antibiotic pretreatment with streptomycin or ampicillin differentially shapes gut microbiota composition. *GsdmD* ^−/−^ mice (C57BL/6 background) were pretreated with either streptomycin (green circles) or ampicillin (red squares), and their gut microbiota composition was analyzed prior to treatment (-1 d p.i.) and 1 d post-treatment (0 d p.i.). (A) Microscopy-based quantification of SYTOX^+^ bacteria per 250 µm^2^. Antibiotic-pretreated mice were assessed 1 d post-treatment. As a control, untreated mice (empty triangles) were added. Data correspond to the same cohort of mice as in Figure 4. (B) Alpha diversity (Shannon index) and (C) principal coordinate analysis (PCoA) based on Bray‒Curtis dissimilarities of individual mice before (untreated) and after treatment (ABX pretreated) with the respective antibiotic. (D) Relative abundances of amplicon sequence variants (ASVs) at the phylum level based on 16S rRNA sequencing. The 6 most abundant phyla are shown, with the rest of the community shown as “Others”. The x-axis indicates the individual mice. In A-C, each data point represents one mouse (A: untreated *n* = 4, streptomycin *n* = 5, ampicillin *n* = 5; B–D: *n* = 4 per group). Panel (A) shows data from one independent experiment, while panels (B–D) show data from a separate independent experiment. Line at the median. The dotted line represents the detection limit. Mann‒Whitney U test (ns – not significant, *P < 0.05).

Next, we sought to investigate how ampicillin and streptomycin pretreatments affect the microbial community composition. *GsdmD* ^*−/−*^ animals were pretreated with a single dose of either streptomycin or ampicillin, and the luminal bacterial density was assessed by fluorescence microscopy. Untreated animals were included as controls to compare luminal bacterial density under antibiotic treatments to baseline levels. Quantitative image analysis revealed that both antibiotic pretreatments substantially reduced total bacterial loads in the gut lumen to a similar extent, resulting in a profound 10- to 20-fold decrease in bacterial density compared to untreated controls (Figure 3A). While fluorescence microscopy-based quantification of luminal bacterial density can be influenced by the uneven spatial distribution of bacteria within the intestinal lumen, we minimized this potential bias by averaging counts across multiple distinct fields of view and independent areas for each mouse. Nevertheless, this approach should be considered an estimate of density rather than an absolute total luminal quantification.

In addition, the gut microbiota composition was analyzed prior to treatment and 1 d post treatment using 16S rRNA gene amplicon sequencing. The 1-day post-antibiotic treatment time point was chosen because it coincides with the standard infection start in our model, thereby reflecting the microbial environment *Salmonella* encounters at the onset of colonization. Hence, we assessed the impact of antibiotics on microbial diversity by measuring alpha diversity (Shannon index) in individual mice before (untreated) and after treatment (antibiotics (ABX) pretreated). Both antibiotics markedly reduced alpha diversity. Relative to their respective baseline values, the reduction was numerically greater in the streptomycin-treated mice (~1.5-fold) than in the ampicillin-treated mice (~1.2-fold), although a minor cage effect was present prior to treatment (Figure 3B). Principal coordinate analysis (PCoA) based on Bray‒Curtis dissimilarity further revealed that the microbial community composition diverged sharply following treatment (Figure 3C). While all the mice clustered together prior to treatment, clear and distinct clustering emerged post-treatment for the ampicillin- and streptomycin-pretreated groups, indicating robust and reproducible shifts in community structure.

At the phylum level, the untreated microbiota were dominated by Bacteroidota (72%), followed by Firmicutes (18%), Verrucomicrobiota (5%), and Proteobacteria (3%) (Figure 3D). Antibiotic treatment substantially reduced Bacteroidota abundance (from 72% to 4%) and shifted community dominance toward Firmicutes (from 18% to 79%). Notably, ampicillin and streptomycin were selected for distinct profiles. Streptomycin pretreatment increased the relative abundances of Actinobacteriota (15% in streptomycin vs 2% in ampicillin) and Desulfobacterota (9% vs 1%), whereas Firmicutes were more abundant in the ampicillin group (66% vs 91%). To assess whether the observed compositional shifts (relative abundances) reflected true changes in bacterial load, we estimated absolute abundances (Figure S3D). For this purpose, we integrated microbiota density measurements (Figure 3A) with relative 16S rRNA gene data (Figure 3D). Because *rrn* copy numbers vary among different bacterial taxa, these integrated values do not reflect absolute cell numbers and are thus expressed as estimated absolute abundance per mL of cecum content. Antibiotic treatment reduced the estimated absolute Bacteroidota and Proteobacteria levels and resulted in Firmicutes-dominated communities. However, the estimated absolute abundances of Firmicutes, Bacteroidota, and Proteobacteria were similar between ampicillin- and streptomycin-treated mice and remained lower than those in untreated controls. In absolute terms, streptomycin increased Actinobacteriota compared to both the untreated and ampicillin-treated groups. Streptomycin-pretreated animals maintained Desulfobacterota at levels comparable to untreated mice, whereas ampicillin led to a reduction in this phylum. Moreover, ampicillin resulted in lower estimated absolute abundances of Verrucomicrobiota compared to streptomycin pretreatment (Figure S3D). At the order level, based on relative abundance data, Coriobacteriales (15% vs 2%) and Oscillospirales (50% vs 6%) were more abundant in streptomycin-pretreated mice, while Erysipelotrichales (9% vs 42%) and the Clostridiales vadinBB60 group (4% vs 37%) were enriched in ampicillin-pretreated animals (Figure S3E).

Taken together, these findings demonstrate that streptomycin and ampicillin pretreatments substantially reduce total bacterial loads in the gut lumen and consistently select for distinct gut microbiota compositions, in agreement with previous studies.^31^ ^,^ ^37^ ^,^ ^38^ We hypothesize that these distinct microbial communities may result in broader differences in the gut environment, such as altered inflammation levels and shifts in nutrient or metabolite availability, which could, in turn, influence subsequent *S*. Tm infection.

### Impact of streptomycin and ampicillin pretreatment on gut metabolites prior to infection

**Figure 4.**
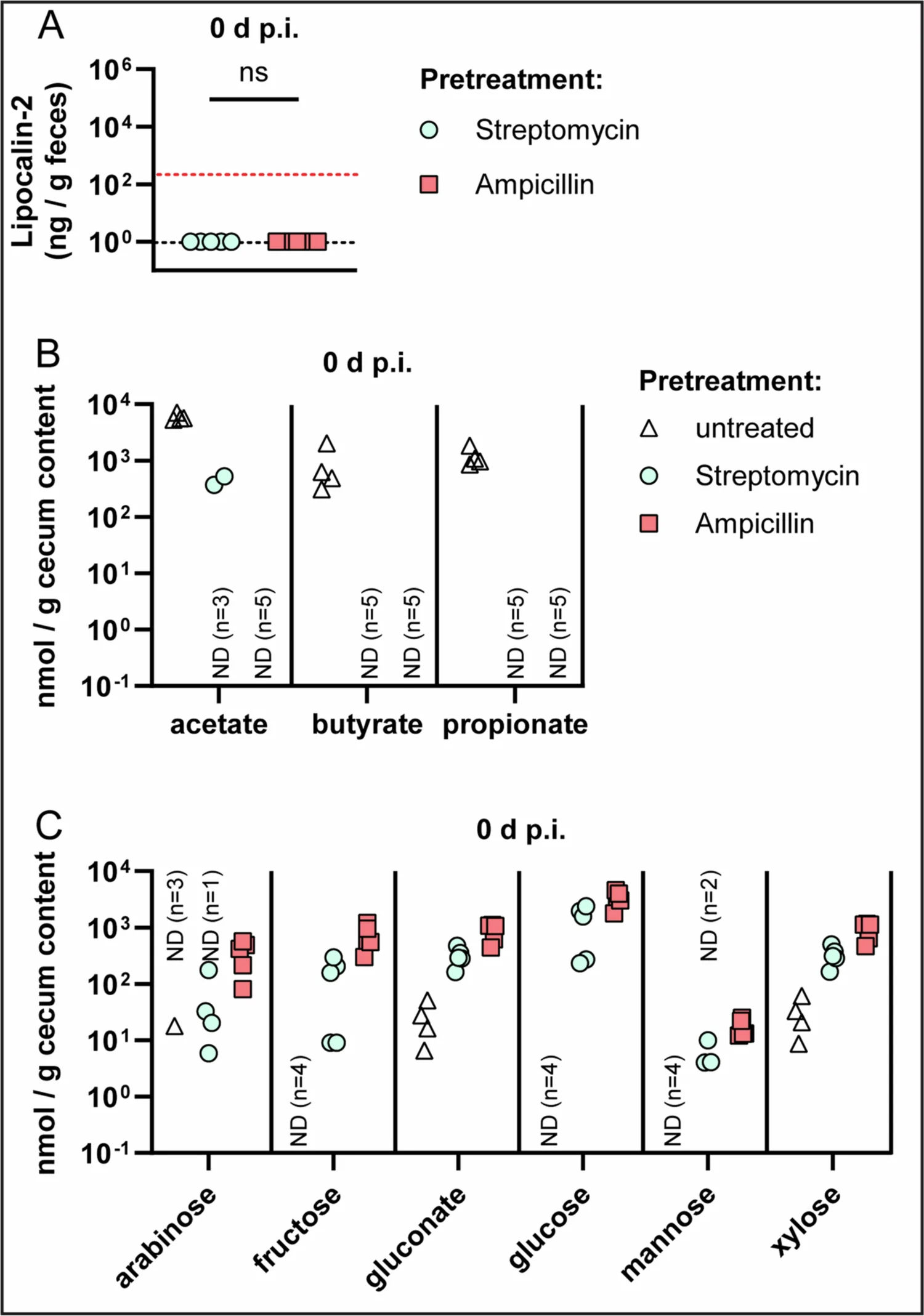
Characterization of antibiotic-specific changes in gut metabolite profiles following pretreatment. *GsdmD* ^−/−^ mice (C57BL/6 background) were pretreated with either streptomycin (green circles) or ampicillin (red squares), and the gut microenvironment was analyzed 1 post-treatment (0 d p.i.). Untreated mice (empty triangles) were added as a control. (A) LCN2 levels (at 0 d p.i.) of cecum content were measured, and all values were below the limit of detection. The red dotted line indicates 200 ng/g feces, the threshold used to define inflammation. (B) LC‒MS-based absolute quantification of the SCFAs acetate, butyrate, and propionate. (C) LC–MS-based absolute quantification of hexoses (glucose, fructose, and mannose), pentoses (arabinose and xylose), and the sugar acid gluconate. The sugar alcohol mannitol was not detected in all the samples. In A–C, each data point represents one mouse (untreated *n* = 4, streptomycin *n* = 5, ampicillin *n* = 5). Data correspond to the same cohort of mice as in Figures 3A and S4A, B. In B–C, ND = not detected; *n* indicates the number of samples below the limit of detection. For statistical testing and calculation of median fold changes, undetected metabolites were imputed with a value corresponding to half of the lowest detected value for the respective metabolite. Statistical comparisons between the streptomycin- and ampicillin-pretreated groups were performed using Mann–Whitney U tests followed by the Benjamini–Hochberg procedure for multiple testing. No metabolites remained statistically significant after FDR correction.

The observed antibiotic-specific changes in luminal microbiota diversity and composition 1 d post-antibiotic treatment suggested that the gut environment at this time point might also differ in other parameters relevant to *S.* Tm colonization. To assess baseline inflammation, we measured LCN2 levels, which were below the detection limit in both treatment groups (Figure 4A), in line with previous observations that antibiotic treatment alone does not cause overt intestinal inflammation.^37^ In parallel, we performed targeted LC‒MS-based metabolomic profiling of cecal contents from untreated and antibiotic-pretreated mice to quantify key metabolites, including amino acids, short-chain fatty acids (SCFAs), sugars, and carbohydrate derivatives, with the aim of characterizing the metabolic landscape 1 d post-antibiotic treatment and determining whether antibiotic-specific microbiota changes were associated with distinct metabolic signatures.

Antibiotic pretreatment with either streptomycin or ampicillin resulted in a marked reduction in all measured SCFAs (acetate, butyrate, and propionate) compared to untreated controls (Figure 4B). In both antibiotic-treated groups, SCFA levels were predominantly below the detection limit, except for two of five streptomycin-pretreated mice with detectable acetate levels of 368 and 527 nmol/g cecum content.

In contrast, the concentrations of most measured amino acids, sugars, and carbohydrate derivatives increased following antibiotic pretreatment (Figures 4C and S4A). Amino acid levels were comparable between streptomycin- and ampicillin-pretreated mice (Figure S4A), whereas several sugars, including arabinose (~21-fold median increase), glucose (~2-fold), fructose (~4-fold), mannose (~3-fold) and xylose (~4-fold) and the sugar acid gluconate (~4-fold), tended to show modestly higher median abundances in ampicillin-pretreated mice relative to streptomycin-pretreated animals (Figure 4C). In addition, organic acid levels were generally comparable between the two pretreatment groups, with the exception of lactate, which was modestly elevated (~2-fold) in the streptomycin-pretreated animals (Figure S4B). However, none of these differences remained statistically significant after FDR correction.

Overall, streptomycin and ampicillin pretreatment caused comparable changes in baseline inflammation and led to marked reductions in SCFAs 1 d post-treatment. Amino acid and organic acid levels showed broadly similar patterns between the two antibiotic regimens, with modest differences observed in selected sugars.

### Early-phase (8 h p.i.) TTSS-1 expression in *S*. Tm^SPI2^ is increased in ampicillin- versus streptomycin-pretreated mice

Although our characterization of metabolite profiles revealed broadly similar patterns following ampicillin and streptomycin pretreatment, the selected metabolites represented only a fraction of the complex intestinal milieu. Other unmeasured microenvironmental factors may still differ between the two conditions and could differentially influence pathogen behavior. We therefore extended our analysis to a functional readout and investigated whether *S*. Tm virulence gene expression differs between the two conditions. Since we used the *S*. Tm^SPI2^ strain, which lacks a functional TTSS-2, we focused on TTSS-1. TTSS-1 is a key virulence factor required for epithelial cell invasion.^1-3^ To assess for differences in *S*. Tm TTSS-1 expression, we transformed *S.* Tm^SPI2^ with a reporter plasmid encoding GFP under the control of the *sicA* promoter,^39^ generating *S*. Tm^SPI2^ p^P*sicAgfp*^. *GsdmD* ^*−/−*^ mice were pretreated with either streptomycin or ampicillin, infected with *S*. Tm^SPI2^ p^P*sicAgfp*^, and GFP expression was analyzed by flow cytometry at 8 h p.i. The gating strategy is provided in Figure S5A.

**Figure 5.**
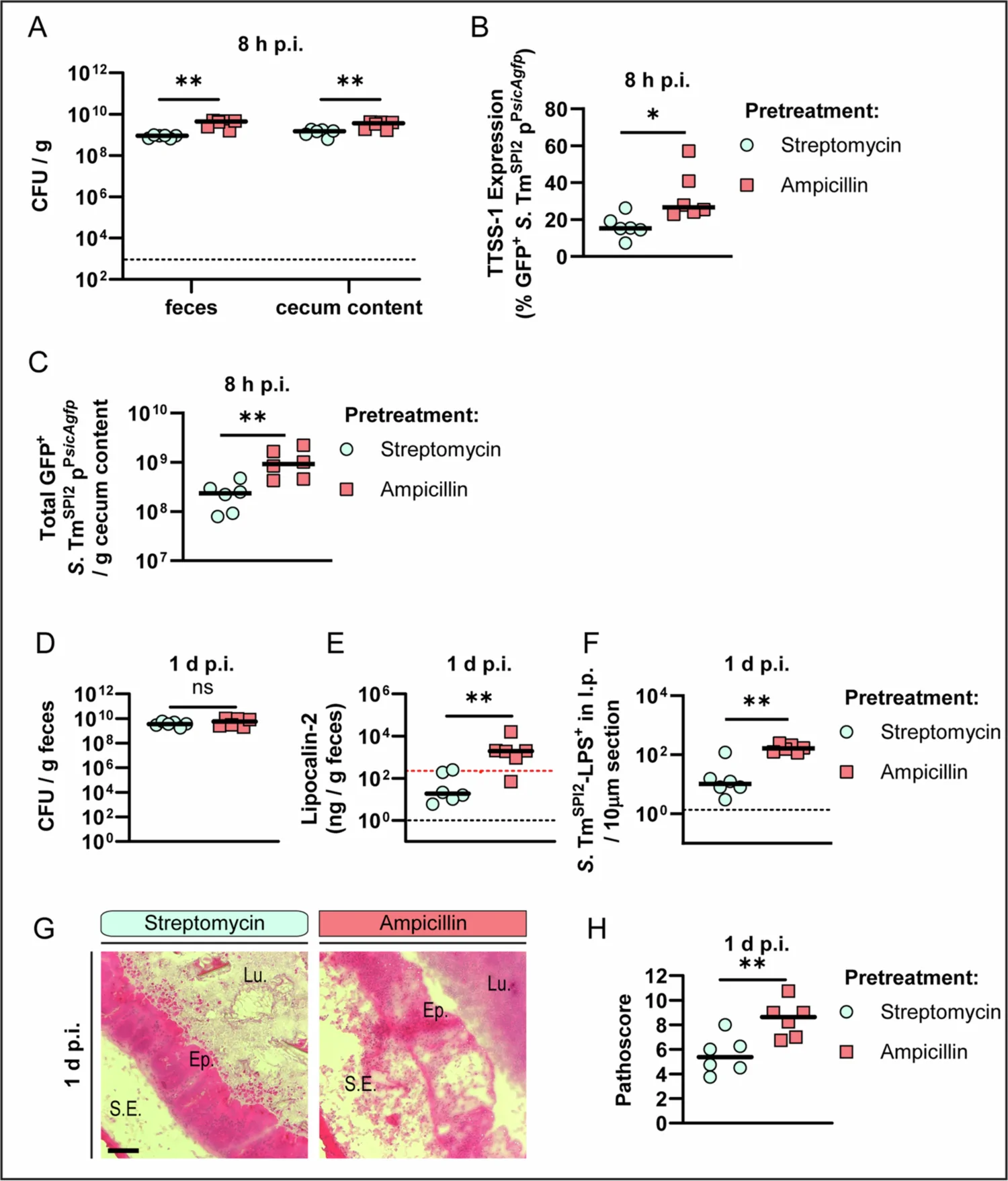
Ampicillin pretreatment is associated with elevated early *Salmonella* TTSS-1 expression, increased mucosal invasion, and elevated inflammation. (A–C) 8 h *S*. Tm^SPI2^ p^P*sicAgfp*^ infection of *GsdmD* ^−/−^ mice (C57BL/6 background) pretreated with either streptomycin (green circles) or ampicillin (red squares). (A) *S*. Tm^SPI2^ p^P*sicAgfp*^ pathogen loads in feces and cecum contents. (B) Fraction of TTSS-1-expressing *S*. Tm^SPI2^ p^P*sicAgfp*^ (GFP^+^) within the total *S*. Tm^SPI2^ p^P*sicAgfp*^ population. The gating strategy is provided in Figure S5A. (C) Total number of TTSS-1-expressing *S*. Tm^SPI2^ p^P*sicAgfp*^ (GFP^+^) per gram of cecum content. (D–H) 1 d *S*. Tm^SPI2^ infection of *GsdmD* ^−/−^ mice (C57BL/6 background) pretreated with either streptomycin (green circles) or ampicillin (red squares). (D) *S*. Tm^SPI2^ pathogen loads in feces. (E) Quantification of inflammation by LCN2 levels of feces. The red dotted line indicates 200 ng/g feces, the threshold used to define inflammation. (F) Microscopy-based quantification of *S*. Tm^SPI2^-LPS^+^ cells in the lamina propria. (G) Representative micrographs of H&E-stained cecal tissue sections at 1 d p.i. Ep. epithelium, Lu. lumen, S.E. submucosa edema. Scale bar: 100 μm. (H) Microscopy-based quantification of histology scores from H&E-stained cecum tissue sections. In A-F and H, each data point represents one mouse (*n* = 6 per group from ≥ 2 independent experiments). Line at the median. The dotted line represents the detection limit. Mann‒Whitney U test (ns – not significant, *P < 0.05, **P < 0.01).

Although the effect size was small, *S*. Tm^SPI2^ p^P*sicAgfp*^ colonization in the feces and cecum contents at 8 h p.i. was significantly higher following ampicillin pretreatment compared to streptomycin (Figure 5A). This finding is consistent with our earlier observations at 1 d p.i. using *S*. Tm^SPI2^ (Figure 1B) and suggests that ampicillin pretreatment is associated with modestly higher initial luminal colonization. In parallel, the fraction of GFP⁺ *S*. Tm^SPI2^ p^P*sicAgfp*^, indicative of TTSS-1 expression, was significantly higher in ampicillin-pretreated mice than in their streptomycin-pretreated counterparts (Figure 5B). Strikingly, the total number of TTSS-1 expressing *S*. Tm (GFP⁺ *S*. Tm^SPI2^ p^P*sicAgfp*^), calculated by multiplying the total CFU by the percentage of GFP^+^ bacteria, was approximately 4-fold higher in ampicillin-pretreated mice, indicating a synergistic effect of increased colonization and higher virulence gene expression (Figure 5C). To confirm the role of TTSS-1 in driving intestinal inflammation in this setup, we infected *GsdmD* ^*−/−*^ mice with an avirulent *S.* Tm strain (*S*. Tm^Δ*invG*Δ*ssaV*^) lacking both functional TTSS-1 and TTSS-2. In this setting, the fecal pathogen loads were comparable between streptomycin- and ampicillin-pretreated groups at day 1 p.i., whereas by day 4 p.i. significantly lower *S*. Tm levels were detected in the streptomycin-pretreated mice (Figure S5B). Despite this late difference in colonization, inflammation levels remained low throughout, and no differences were observed between the streptomycin- and ampicillin-pretreated groups (Figure S5C), suggesting that the observed inflammatory differences are indeed TTSS-1-dependent.

Since TTSS-1 is essential for epithelial cell invasion, we next asked whether the differences in TTSS-1 expression observed during the early phase of infection (8 h p.i.) would translate into differences in host tissue colonization levels at 1 d p.i. Specifically, we hypothesized that bacterial loads in the cecum tissue, representing the primary site of invasion in this model, would be higher in ampicillin-pretreated animals. To test this, *GsdmD* ^*−/−*^ mice were pretreated as described above and infected with *S*. Tm^SPI2^ for 1 d. We used *S*. Tm^SPI2^ instead of the reporter strain *S*. Tm^SPI2^ p^P*sicAgfp*^ to avoid any potential influence of the reporter plasmid on bacterial virulence. While no statistically significant differences in luminal colonization were observed between the two pretreatment groups, LCN2 levels were ~100-fold higher in ampicillin-pretreated mice (Figure 5D,E), in line with previous results (Figure 1C). Notably, fluorescence microscopy revealed a ~10-fold increase in *S*. Tm^SPI2^ cells positive for LPS staining (*S*. Tm–LPS⁺) within the lamina propria of ampicillin-pretreated mice, potentially reflecting elevated early TTSS-1 expression (Figure 5F). We focused our analysis on the lamina propria because LPS staining does not allow us to reliably distinguish between bacteria that have invaded epithelial cells and those that are merely attached to the epithelial surface. Thus, quantifying *S*. Tm–LPS⁺ cells within the lamina propria serves as a more robust indicator of successful tissue invasion. This increase in mucosal invasion was accompanied by significantly elevated histopathology scores (Figure 5G,H). Notably, histopathology scores in the ampicillin group at 1 d p.i. had already reached levels comparable to those observed at 4 d p.i., whereas scores in the streptomycin-pretreated mice declined between 1 d and 4 d p.i., possibly due to the regrowth of the microbiota and partial restoration of colonization resistance (Figures 1D,E and 5G,H).

Together, these findings suggest that ampicillin pretreatment is associated with modestly higher initial luminal colonization by *Salmonella* and elevated early TTSS-1 expression (8 h p.i.), which correlates with increased mucosal invasion and inflammation observed at 1 d p.i. Importantly, while ampicillin is clearly associated with elevated TTSS-1 expression, the specific trigger – whether microbial, metabolic, or an altered luminal condition – underlying this higher expression remains unidentified. Nonetheless, the absence of these effects in infections with TTSS-1- and TTSS-2-deficient *S.* Tm supports a key role for TTSS-1 in mediating these differences.

### Antibiotic-dependent effects on intestinal inflammation are maintained in *S*. Tm^WT^ infection

**Figure 6.**
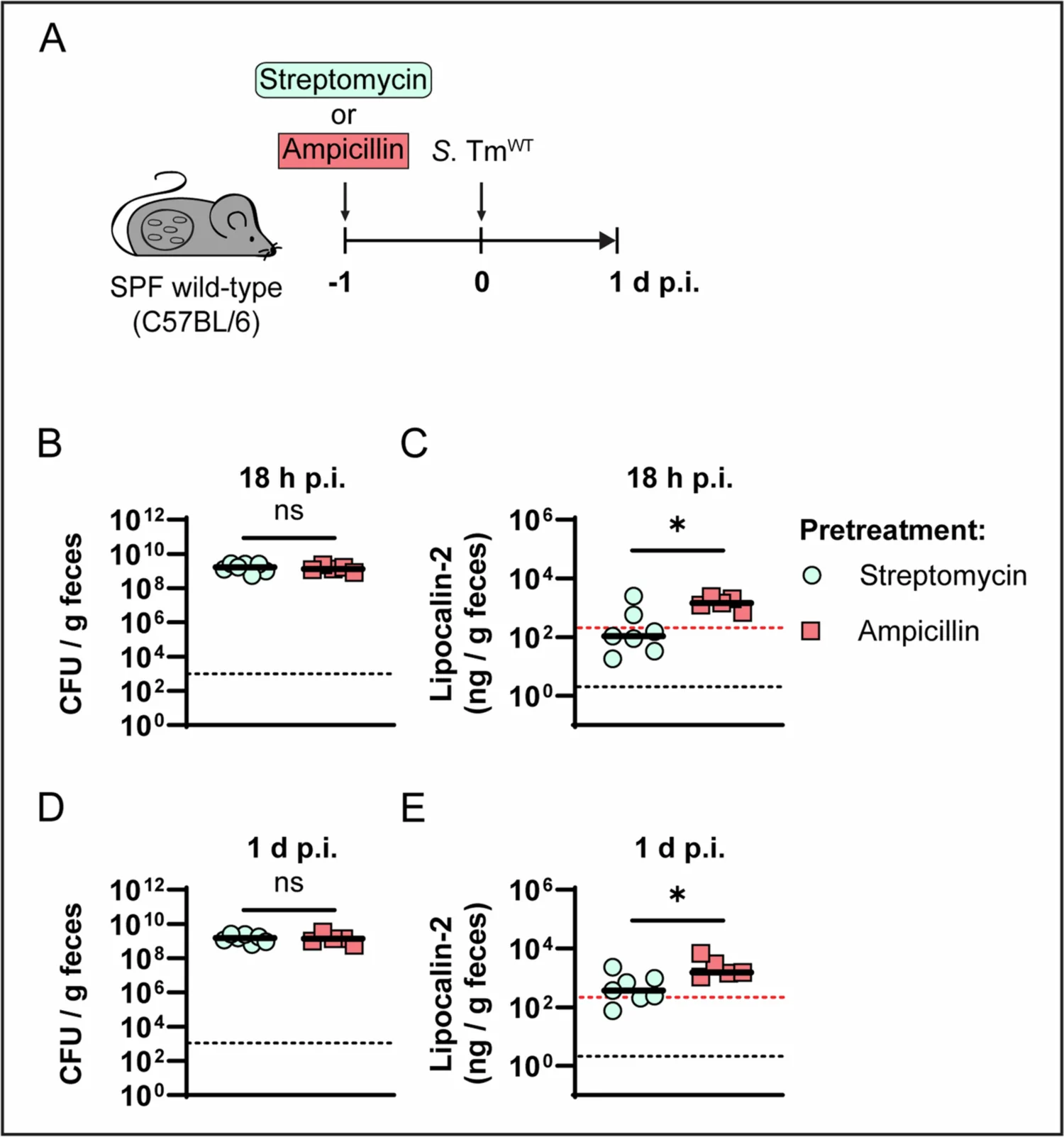
Ampicillin pretreatment results in stronger intestinal inflammation than streptomycin pretreatment during *S*. Tm^WT^ infection in WT mice. 1 d *S*. Tm^WT^ infection of WT mice (C57BL/6) pretreated with either streptomycin (green circles) or ampicillin (red squares). (A) Experimental setup. (B) *S*. Tm^WT^ pathogen loads in feces at 18 h p.i. (C) Quantification of inflammation by LCN2 levels of feces at 18 h p.i. The red dotted line indicates 200 ng/g feces, the threshold used to define inflammation. (D) *S*. Tm^WT^ pathogen loads in feces at 1 d p.i. (E) Quantification of inflammation by LCN2 levels of feces at 1 d p.i. The red dotted line indicates 200 ng/g feces, the threshold used to define inflammation. In B-E, each data point represents one mouse (streptomycin *n* = 7, ampicillin *n* = 5, from ≥ 2 independent experiments). Line at the median. The dotted line represents the detection limit. Mann‒Whitney U test (ns–not significant, *P < 0.05).

After identifying TTSS-1 expression as an important parameter that may explain the differences between streptomycin- and ampicillin-pretreated mice, we next asked whether these observations were specific to our experimental system or more broadly applicable. To address this, mice from our standard WT C57BL/6 colony, which harbors a complex SPF gut microbiota, were pretreated with streptomycin or ampicillin prior to infection with *S.* Tm^WT^ for 1 d (Figure 6A). We focused on 18 h p.i., as previous work had indicated that slight differences in the mucosal inflammation kinetics elicited by *S.* Tm^WT^ can be robustly detected at this time point.^40^

At 18 h p.i. and at 1 d p.i., fecal pathogen loads were comparable between groups, whereas LCN2 levels were consistently elevated in ampicillin-pretreated mice (Figure 6B–E), indicating enhanced intestinal inflammation. Notably, although LCN2 levels remained significantly different between the groups at 1 d p.i., the magnitude of this difference was less pronounced than at 18 h p.i. This observation is consistent with the established kinetics of *S*. Tm^WT^-induced intestinal inflammation in antibiotic-pretreated mice. Because *S*. Tm^WT^ expresses a functional TTSS-2, and the increasing contribution of TTSS-2-dependent virulence enhances intestinal inflammation over time,^41^ which may reduce the relative difference in LCN2 levels between the groups.

Overall, these findings in WT mice infected with *S*. Tm^WT^ are consistent with our observations using attenuated *S*. Tm strains in both WT and *GsdmD* ^*−/−*^ mice (Figures 1 and S1), demonstrating that the antibiotic-dependent effects on gut inflammation are not restricted to attenuated bacteria or specific host genotypes, suggesting that antibiotic pretreatment broadly shapes intestinal inflammation during *S*. Tm infection.

## Discussion

In this study, we compared the effects of streptomycin and ampicillin pretreatment on *S.* Tm infection in mice and found that the choice of antibiotic critically shapes infection outcome. Both regimens are commonly used to disrupt colonization resistance, and both facilitated luminal *Salmonella* colonization. However, they were associated with distinct infection kinetics, pathogen behavior, and host responses. Ampicillin-pretreated mice developed strong and sustained inflammation, whereas streptomycin-pretreated mice showed milder responses. These differences were observed across different host backgrounds (C57BL/6 and 129S6/SvEvTac mice) and in infections with both *S*. Tm^SPI2^ and *S*. Tm^WT^, were independent of host GSDMD status but were linked to pronounced antibiotic-specific microbiota shifts. Consistently, GF mice pretreated with streptomycin or ampicillin showed no differences in infection outcome, supporting a microbiota-dependent mechanism. In addition, we performed a targeted characterization of the luminal metabolic landscape 1 d post-treatment, which revealed subtle differences in metabolite profiles. Notably, ampicillin pretreatment was associated with elevated early TTSS-1 expression and increased mucosal invasion. Together, our findings indicate that the specific antibiotic used for pretreatment can fundamentally shape infection dynamics.

Antibiotic pretreatment can influence infection outcomes through multiple effects on the microbiota, host, pathogen, and their interactions.^17^ ^,^ ^18^ ^,^ ^31^ ^,^ ^37^ Because antibiotics primarily target the microbiota, their most immediate impact is likely exerted at this level, with downstream consequences for the luminal environment encountered by the pathogen. Although streptomycin (an aminoglycoside) and ampicillin (a *β*-lactam) both act bactericidally and reduce the overall microbiota density to a similar extent, they generated distinct community compositions in our model (Figure 3). This could potentially be explained by their differential mode of action, which may variably affect specific bacterial taxa, thereby resulting in altered community structure consistent with previous reports of class-specific effects of antibiotics on microbiota composition.^31^ ^,^ ^42^ ^,^ ^43^ Despite comparable reductions in alpha diversity and total bacterial load, ampicillin pretreatment resulted in faster luminal *Salmonella* expansion and stronger inflammation. This suggests that infection outcomes are not primarily determined by overall diversity or bacterial density, but rather by the specific microbial communities that persist or re-establish after treatment. In streptomycin-pretreated mice, the residual microbiota may limit *Salmonella*-induced inflammation, for example, by competing for nutrients, constraining virulence expression, or dampening host inflammatory responses. Previous work identified *Mucispirillum schaedleri* as protective in this context,^44^ but Deferribacterales were not detected in the majority of samples by 16S rRNA analysis and, when present, were observed only at very low relative abundances, arguing against a major contribution here. Instead, streptomycin pretreatment enriched Coriobacteriales and Oscillospirales, the latter also being elevated in mice that had recovered from long-term *S.* Tm^SPI2^ infection.^45^ Notably, members of the Coriobacteriales are capable of producing lactate,^46^ which may contribute to the modestly elevated lactate levels observed in streptomycin- compared to ampicillin-pretreated mice (Figure S4B). In contrast, ampicillin pretreatment enriched the Erysipelotrichales and Clostridiales vadinBB60 group, taxa that are thus associated with more pro-inflammatory environments or heightened pathogen virulence. Determining whether these taxa actively shape *Salmonella*-induced inflammation will require future mechanistic work.

Streptomycin and ampicillin pretreatment generated distinct microbiota compositions that coincided with differences in early TTSS-1 expression. Because TTSS-1 expression is known to be regulated by environmental cues, including microbial metabolites such as SCFAs,^47-49^ we examined the luminal metabolite landscape under both pretreatment regimens. Although TTSS-1 expression responds to SCFAs such as butyrate, propionate, and acetate,^47^ ^,^ ^49-54^ SCFA concentrations were predominantly below the detection limit under both antibiotic regimens (Figure 4B), preventing a reliable comparison between groups and arguing against a major role for SCFAs in mediating the differential virulence expression observed here. Notably, this decline in SCFA concentrations to below the limit of detection following both antibiotic treatments represents a substantially greater reduction than would be expected from the observed 10- to 20-fold decrease in total bacterial density alone (Figure 3A). While the accumulation of unconsumed nutrients, such as sugars and amino acids (Figures 4C and S4A), can be partly attributed to reduced microbial biomass, the disproportionate loss of fermentation products suggests a more profound disruption of microbial function. One potential explanation is the depletion of key SCFA-producing taxa. In particular, members of the order Bacteroidales, which includes major propionate producers utilizing the succinate pathway,^55^ were strongly reduced under both antibiotic regimens, consistent with the concurrent loss of propionate production (Figures 4B and S3E). In addition, the surviving microbiota may still have remained metabolically suppressed from the acute antibiotic stress. Consequently, lactate accumulated in antibiotic-pretreated mice relative to untreated controls, despite normally being rapidly converted into SCFAs in the healthy gut lumen.^56^ Collectively, the loss of shared key fermentative taxa, reduced bacterial biomass, and community-wide metabolic inactivity may explain why streptomycin- and ampicillin-pretreated mice displayed broadly similar metabolite profiles despite their distinct 16S-based community structures.

Moreover, several sugars were observed at modestly higher levels in ampicillin- compared to streptomycin-pretreated mice prior to infection. Notably, D-glucose and D-fructose support *S.* Tm colonization across diverse microbiota contexts,^57^ and L-arabinose can likewise promote pathogen expansion by providing a competitive advantage in the gut.^58^ ^,^ ^59^ Recent work by Yu et al. further demonstrated that sugars such as L-arabinose accelerate *S*. Tm growth kinetics *in vitro* and boost early-stage pathogen expansion in streptomycin-pretreated mice.^60^ Together, these findings suggest that increased sugar availability in ampicillin-pretreated mice may contribute to the increased early colonization observed in this model. At first glance, the elevated levels of L-arabinose observed here seem difficult to align with the increased TTSS-1 expression, as L-arabinose has previously been shown to repress TTSS-1 expression *in vitro.* ^60^ ^,^ ^61^ However, recent *in vivo* evidence suggests that increased L-arabinose availability paradoxically correlates with a substantial elevation of early-stage TTSS-1 signals due to accelerated pathogen growth and population expansion.^60^ We do not interpret these findings to suggest that L-arabinose directly drives TTSS-1 activation in ampicillin-pretreated mice, as the gut environment contains a complex mixture of host- and microbiota-derived signals that may collectively shape virulence responses. Rather, these observations demonstrate that our observation of concurrent sugar enrichment, robust early colonization, and elevated TTSS-1 expression is entirely compatible with recent *in vivo* findings. Whether faster population growth additionally permits the maintenance of a larger subpopulation of slower-growing TTSS-1⁺ cells while overall pathogen expansion is sustained requires further investigation.^39^ ^,^ ^60^ Because the sugar differences detected here reflect relatively modest endogenous shifts rather than the high-level supplementation used in the previous study, further experiments are needed to determine whether these specific physiological concentrations are sufficient to produce comparable effects. Furthermore, it should be noted that while our metabolomics data characterize the baseline nutritional environment, they revealed no specific candidate metabolite linked to this phenotype. Consequently, the precise molecular basis underlying the elevated TTSS-1 expression observed in ampicillin-treated mice remains unresolved.

Antibiotics may also influence infection dynamics through host-directed mechanisms, such as acting directly on host tissues or indirectly through microbiota-mediated effects, such as changes in epithelial physiology, mucus thickness, or immune activity.^29^ ^,^ ^38^ ^,^ ^62^ ^,^ ^63^ However, baseline LCN2 levels 1 d after pretreatment were below the detection limit in both groups, arguing against overt antibiotic-induced inflammation occurring at this time point (Figure 4A). Consistently, germ-free mice pretreated with streptomycin or ampicillin and subsequently infected showed no differences in infection outcome, further supporting a microbiota-dependent rather than a host-directed mechanism (Figure S3A–C). Moreover, infection with a TTSS-1- and TTSS-2-deficient strain (*S*. Tm^Δ*invG*Δ*ssaV*^) did not trigger elevated LCN2 levels, indicating that the heightened inflammation observed in ampicillin-pretreated mice depends on pathogen virulence and is not solely driven by antibiotic-induced microbiota perturbation (Figure S5B,C). Finally, while we cannot fully exclude that residual antibiotics in the gut may have influenced pathogen growth or virulence gene expression, the available data suggest that this effect is unlikely to be the primary driver of the observed differences.

Differences between pretreatments also became apparent later during infection. In the streptomycin-pretreated animals, the luminal *S.* Tm^SPI2^ densities declined toward the end of infection, accompanied by decreasing histopathology scores and reduced LCN2 levels between day 1 and day 4 p.i. (Figures 1 and 5G,H), which could be consistent with a partial recovery of the gut microbiota.^45^ In contrast, in the ampicillin-pretreated animals, the pathogen loads remained at the carrying capacity, the histopathology scores remained high, and the LCN2 levels did not decline, suggesting impaired microbiota recovery. During *S.* Tm^WT^ infection with streptomycin pretreatment, such a decline in CFU counts is typically not observed, likely because additional inflammation driven by TTSS-2 activity prevents microbiota regrowth.^24^ ^,^ ^32^ The lack of recovery in ampicillin-pretreated mice could therefore be explained by the stronger and more sustained inflammation observed in this setting. However, a comparable difference in CFU counts between the two antibiotics was also present during infection with an avirulent strain lacking TTSS-1 and TTSS-2 (Figure S5B, C), where inflammation is minimal, indicating that pathogen-induced inflammation alone cannot account for the impaired recovery after ampicillin pretreatment. Antibiotics themselves could, in principle, induce subtle host alterations that favor pathogen persistence, but baseline LCN2 levels and LCN2 dynamics during avirulent infection were comparable between groups, arguing against a major inflammation-driven effect of the antibiotic alone. Together, these observations suggest that additional, inflammation-independent consequences of ampicillin treatment may contribute to the lack of microbiota recovery. Ampicillin may more effectively eliminate key commensal competitors of *S*. Tm, thereby reducing ecological pressure on the pathogen, or may alter host factors such as mucus production in ways that limit commensal re-establishment.

Overall, ampicillin pretreatment resulted in more stable luminal colonization and consistently higher inflammation throughout infection, reducing variability between animals. This makes ampicillin-pretreated mice a useful model for studying attenuated *Salmonella* strains such as *S*. Tm^SPI2^, where TTSS-2, an important driver of late-stage *Salmonella*-induced inflammation,^24^ is missing. Importantly, ampicillin pretreatment also minimizes the impact of confounding factors such as differences in microbiota composition between mouse lines, which can otherwise influence experimental readouts. At the same time, our findings highlight that different antibiotic pretreatments generate distinct intestinal environments that are associated with differences in infection kinetics, pathogen behavior, and host responses. Although we do not establish causality, our data indicate that the observed phenotypes likely arise from a dynamic interplay between the microbiota, the host, and the pathogen. The results obtained under different pretreatment regimens should therefore not be directly compared without considering these contextual differences. More broadly, our work underscores that the choice of the specific antibiotic used for pretreatment is a critical experimental variable in enteric infection models and should be carefully matched to the biological question being addressed.

## Materials and methods

### Mouse lines

Male and female C57BL/6 mice with different microbiota complexity (germ-free (GF) and specific pathogen-free (SPF)) and SPF 129S6/SvEvTac strains were kept in individually ventilated cages of the ETH Zürich mouse facility (EPIC and RCHCI). C57BL/6 wild-type mice (The Jackson Laboratory) and *GsdmD* ^*−/−*^ mice,^64^ as well as 129S6/SvEvTac mice (The Jackson Laboratory), between 8 and 15 weeks old, were used for the experiments. Genotyping was done by PCR. The sample size was not predetermined, and the mice were randomly allocated to groups.

### Study design

The experimental unit was the individual mouse. Where available, littermates were used to minimize genetic and environmental variability. Mice were housed in separate cages by the experimental group, with the animals from multiple independent experiments contributing to each experimental group. While formal randomization of cage rack position and sample processing sequence was not explicitly performed, mice were housed in the same room under standardized environmental conditions to minimize potential confounders. All experimental groups were handled, sampled, and processed identically within each independent experiment. Investigators were aware of the experimental groups during the conduct of the experiments and data analysis. Histopathological scoring, microscopy-based image analysis, ELISA measurements, and qPCR analyses were performed by investigators blinded to the experimental groups.

### Bacterial strains

This study used mutant derivative bacterial strains of *Salmonella* Typhimurium SL1344. *S*. Tm^SPI2^ p^P*sicAgfp*^ was generated by transforming M2730 with the reporter plasmid pM974, which encodes GFP under the control of the *sicA* promoter.^39^ The desired transformant was selected on antibiotic-containing media. All the strains used in this study are summarized in Table S1.

### Infection experiments

The mice were pretreated 24 h prior to infection with a single fixed dose of either 25 mg streptomycin sulfate (Sm; Cat# A1852; PanReac AppliChem) or 20 mg ampicillin sodium salt (Amp; Cat# A0839; PanReac AppliChem). Both antibiotics were dissolved in sterile water and administered in a total volume of 50 µL per mouse via oral gavage. These antibiotic doses were selected based on established protocols.^20^ ^,^ ^32^ ^,^ ^40^ Following pretreatment, the mice were infected at 0 h with 5 × 10^7^ CFU *S*. Tm by oral gavage. *S.* Tm strains were grown overnight in lysogeny broth supplemented with Sm (50 µg/ml) and Amp (100 µg/ml) at 37 °C, followed by sub-culturing at a 1:20 ratio in antibiotic-free lysogeny broth for 4 h. The resulting inoculum was washed and resuspended in 50 µL of sterile PBS (Cat# 3-05F29-I; BioConcept). The mice were monitored daily, and feces were collected at the indicated time points in pre-weighed tubes containing PBS. At the designated endpoints, the mice were euthanized by ${\text{CO}}_{2}$ asphyxiation using a slow stream of ${\text{CO}}_{2}$ , with death confirmed by major organ removal. The organs were harvested at the end of each experiment. The samples were homogenized with a steel ball in a tissue lyser (Qiagen) for 2.5 min at 25 Hz frequency. For cecum tissue plating, the content was removed, and the sample was washed briefly in PBS before being incubated for 60‒90 min in 500 µl PBS/400 µg/ml gentamycin (Cat# A1492; PanReac AppliChem) to remove extracellular bacteria, and was washed extensively in 1 ml PBS (3 × 30 seconds) before plating. Bacterial burdens were analyzed by CFU plating assays on MacConkey agar (Oxoid) supplemented with 50 µg/ml Sm or 100  µg/ml Amp. All animal experiments were approved by the Kantonales Veterinäramt Zürich (licenses ZH158/2019, ZH108/2022, ZH109/2022, ZH092/2025, and ZH140/2025) and reported in compliance with the ARRIVE guidelines. Humane endpoints and exclusion criteria for severe distress (apathy, ruffled fur, hunched posture, and severe diarrhea) were established *a priori*. Because no animals reached these predefined endpoints, no animals or data points were excluded from the final analysis. A total of 158 mice were used across all the experiments in this study.

### Microbiota transfer by co-housing

Germ-free wild-type female mice (recipients) were co-housed with conventionally raised SPF *GsdmD* ^*−/−*^ female mice (donors) for at least 4 weeks to allow horizontal transfer and stable establishment of the donor microbiota. The donor-to-recipient ratios ranged from 1:1 to 2:3, depending on cage availability. Following this period, the mice remained co-housed and were subjected to either Sm or Amp pretreatment prior to oral *S*. Tm infection.

### Immunofluorescence microscopy

Cecal tissue was fixed in 4% paraformaldehyde (Cat# 158127; Sigma–Aldrich), saturated in PBS/20% sucrose (Cat# CLM-9811-PK; Eurisotop), and snap-frozen in optimal cutting temperature compound (Cat# 4583; Tissue-Tek). Frozen cecal tissues were sectioned at 10 μm using a microtome (Microm HM525, Thermo Scientific) and mounted on glass slides (Superfrost++, Thermo Scientific). Air-dried sections were outlined with an ImmEdge Hydrophobic Barrier Pen (Cat# H-4000; Vector Laboratories), rehydrated in PBS (5 min), permeabilized with PBS/0.5% Triton X-100 (Cat# X100; Sigma–Aldrich) (10 min), and blocked in PBS/10% normal goat serum (NGS; Cat# S-1000; Reactolab SA) (≥30 min). The antibodies used for staining included *α*-*S.* Tm LPS (O-antigen group B factor 4–5, Difco; 226601, RRID: AB_3676217) (1:400) and *α*-EpCam/CD326 (clone G8.8; Biolegend) (1:200) in combination with suitable secondary antibodies (*α*-rabbit-Alexa-Fluor488; Abcam Biochemicals; #ab150077, RRID: AB_2630356) (1:200); *α*-rat-Cy3 (Jackson; #112-165-167, RRID: AB_2338251) (1:200) and fluorescent probes, such as Sytox Green nucleic acid stain (Invitrogen; S7020; 0.1 μg/ml final concentration), AlexaFluor647-conjugated Phalloidin (Molecular Probes; A22287) (1:1000) and DAPI (Sigma–Aldrich; D9542) (1:1000). The samples were incubated with primary antibodies in 10% NGS/PBS (≥40 min), washed in PBS (3×), followed by incubation with secondary antibodies and fluorescent probes (≥40 min), washed again (3×), and mounted with Mowiol (Cat# 475904-100; VWR International AG) and a coverslip.

Microscopy analysis was done with confocal fluorescence microscopy (Zeiss Axiovert 200 M microscope) and widefield fluorescence microscopy (Axioplan 2 imaging microscope). Images were processed using VisiView (Visitron) and Fiji (ImageJ). For quantification of luminal bacterial density, confocal images were taken, and luminal bacteria were manually counted in 3 areas (250 μm^2^ each) per image, across 3 images per mouse.

### Histology

Cecal tissue was embedded in optimal cutting temperature compound, snap-frozen, and sectioned at a thickness of 5 μm. The sections were subsequently air-dried and subjected to hematoxylin (Cat# 51275; Sigma–Aldrich) and eosin (Cat# HT1103128; Sigma–Aldrich) staining. Histopathological assessment was conducted in a blinded fashion according to established protocols.^17^ A scoring system was used, where 0 indicated normal intestinal morphology with no inflammation, scores of 1–2 indicated minimal inflammatory changes, 3–4 indicated mild inflammation, 5–8 corresponded to moderate inflammation, and 9–13 reflected severe inflammation.

### Lipocalin-2 ELISA

Fecal LCN2 levels were assessed using a commercial ELISA kit (DY1857; R& D Systems) according to the manufacturer’s instructions. In summary, the fecal pellets were homogenized in PBS, centrifuged at 16,000 × *g* for 5 min, and the supernatants were undiluted or diluted (1:20 or 1:400) for LCN2 measurement. The absorbance was measured at 405 nm using the Spectramax Plus instrument (Molecular Devices). Concentrations were calculated using a four-parameter logistic (4PL) curve in GraphPad Prism version 8.

### 16S analysis

Fecal pellets were collected and stored at −20 °C. Genomic DNA was extracted using the AllPrep DNA/RNA Mini Kit (Cat# 80204; Qiagen) with bead-beating-based disruption of bacterial membranes. The DNA concentration was quantified by Qubit, and 5 ng was used for library preparation. The V4 region of the 16S rRNA gene was amplified with the primers 515f and 806r using a two-step PCR approach with Q5 High-Fidelity DNA Polymerase (Cat# M0491L; New England Biolabs). Amplicons (~450 bp) were purified, indexed, pooled equimolarly, and sequenced on the Illumina MiSeq platform (2 × 300 bp).

Raw sequencing reads from all samples served as input for the inference of ASVs using dada2 v1.22.^65^ Primer sequences were removed using cutadapt v3.5,^66^ and only paired reads that contained both primers (*-O 12, --discard-untrimmed, --minimum-length 75*) were kept for downstream analysis. Next, the reads were quality filtered using the filterAndTrim function of the dada2 v1.22^65^ package (*matchIDs = FALSE, rm.phix = TRUE, maxEE = 2, truncQ = 2, minLen = 141, truncLen = c(191, 151)*). The *learnErrors* function (*nbases = 1e7, randomize = TRUE, errorEstimationFunction = loess_error_function_mod*) was used to calculate error estimates for nucleotide transition probabilities. To infer the ASVs, the function *dada* was used to calculate sample inference using *pool = pseudo* as a parameter.

Reads were merged using the *mergePairs* function, and bimeras were removed with the *removeBimeraDenovo* (*method = pooled*). The taxonomic annotation was performed using dada2 assignTaxonomy function and Silva v138^67^ as a database.

### qPCR analysis of the total 16S rRNA genes

Total genomic DNA was extracted from fecal pellets using the DNeasy PowerSoil Pro Kit (Cat# 47016; Qiagen) according to the manufacturer’s instructions. To ensure uniform input across all samples, the final DNA elution volume was volumetrically adjusted with sterile water to achieve a standardized ratio of 0.1g of fecal mass per mL. The total bacterial biomass was subsequently evaluated by qPCR using the primers 515f and 806r targeting the V4 region of the bacterial 16S rRNA gene. Amplification reactions were performed in a 20  µl volume containing FastStart Universal SYBR Green Master (Cat# 4913914001; Roche) and 1  µl of the normalized DNA template. Reactions were run on a QuantStudio 7 Flex Real-Time PCR System (Applied Biosystems, Life Technologies). Ct values were recorded, and samples failing to amplify after 40 cycles were assigned a baseline value of Ct = 40, representing the assay's limit of detection.

### Metabolite measurements

LC‒MS analyses were carried out with an Ultimate 3000 ULPC instrument (Thermo Scientific, Waltham, MA, USA) hyphenated to a QExactive plus mass spectrometer (Thermo Scientific, Waltham, MA, USA).

#### C18 reversed-phase analysis of hydrazones from short-chain fatty acids, sugars, and sugar acids

For the analysis of SCFAs and other carboxylates, samples were mixed with a corresponding stable isotope labeled standards mixture and derivatized by the addition of 3-Nitrophenylhydrazine (3-NPH·HCI) (Cat# QB-0416; Combi-Blocks) and *N*-(3-dimethylaminopropyl)-N′-ethylcarbodiimide hydrochloride (EDC·HCI) (Cat# 03449; Sigma–Aldrich) in 50% aqueous acetonitrile (Cat# A955-212; Fisher Chemical) to form their hydrazones. After incubation at 40 °C for 45  min with mixing, the reactions were quenched with trifluoroacetic acid (Cat# T/3256/PB08; Fisher Chemical). After dilution with 70% isopropanol (Cat# 34965-1L; Sigma–Aldrich) in water (Cat# W6-212; Fisher Chemical), the samples were centrifuged and analyzed (final concentration: 25  µg cecum content/ml).

Sugars and sugar acids were analyzed separately. Again, samples were mixed with a mixture of corresponding stable isotope-labeled standards, and the same protocol as described above was applied, except that 6% pyridine (Cat# 11390438; Thermo Scientific Chemicals) was added to the EDC solution.

To analyze hydrazones, we applied a previously described method^68^ with slight modifications. LC separation was performed using a Kinetex XB C18 column (particle size 1.7 µm; 50 × 2.1 mm, Phenomenex, Torrance, CA, USA) with 0.1% formic acid (Cat# 10596814; Fisher Chemical) in water (solvent A) and 0.1% formic acid in acetonitrile as the mobile phases. Sample injection volume was 2 µl. At a flow rate of 500 µl min^−1^, a linear gradient was applied with solvent B changing as follows: 0 min: 2%; 6 min: 95%; 8 min: 95%; 8.3 min: 2%. Subsequently, the column was equilibrated for 2 min at the initial condition. MS analysis was done in the negative FTMS mode at a mass resolution of 70,000 (at m/z 200) applying the following source parameters: spray voltage: −2.7 kV; S-lens RF level: 50; sheath gas: 50; aux gas: 20; sweep gas: 0; and aux gas heater: 350 °C. For details, see S2 Table.

#### HILIC analysis of native compounds

For LC separation of all proteinogenic amino acids with the exception of glycine, as well as the pentoses xylose and arabinose, we applied a previously described method^69^ with some modifications. In brief, we used an AQUITY BEH NH2 column (particle size 1.7 µm; 100 × 2.1 mm, Waters, Milford, MA, USA) with a buffer consisting of 10 mM ammonium formate (Cat# 70221-25G-F; Sigma–Aldrich) (short buffer) in water:acetonitrile (50:50) (solvent A) and the same buffer in acetonitrile:water:methanol (Cat# A456-212; Fisher Chemical) (95:5:5) as the mobile phases. To shift pH to 1.76 ml of formic acid was added to both mobile phases. The sample injection volume was 2 µl. At a flow rate of 500 µl min^−1^, a linear gradient was applied with solvent B changing as follows: 0 min: 84.3%; 1.5 min: 84.3%; 5.5 min: 5.3%; 7.5 min: 5.3%; 8 min: 84.3%, 10 min: 84.3%. Heated electro spray ionization was performed applying the following source parameters: spray voltage – 2.7 kV, +3.4 kV; S-lens RF level: 50; sheath gas: 50; aux gas: 20; sweep gas: 0; aux gas heater: 350 °C. Unlabeled and labeled isotopologues of all analytes were measured by parallel reaction monitoring, with the exception of alanine and serine, which were measured in the FTMS mode. When applying parallel reaction monitoring, precursor isolation window width was set to 1.5 Da, and the fragment ions were measured at a mass resolution of 17600 (at m/z 200). For details, see Table S3.

#### Quantification

For absolute quantification of the measured compounds, isotope dilution mass spectrometry was applied. To this end, samples and standards were spiked with the same heavy isotope-labeled internal standard mixtures. Calibration curves were calculated from compound peak area ratios (standard: labeled standard) of standard dilution series measured in triplicate by applying weighted linear least square fitting (Tables S4 and S5). To calculate the compound concentrations from the peak area ratios in samples, the inverse fitting curve function $x=\;\frac{y-b}{a}$ was applied. Mock samples were subjected to the same extraction and LC–MS workflow and used for background subtraction.

### Bacterial flow cytometry

The activity of the *sicA* promoter upon infection was analyzed using a plasmid-based P*sicA-gfp* reporter.^39^ Cecum content was collected in 1  ml of PBS and homogenized. After centrifugation (500 × g; 1 min), the supernatant was collected, washed, and resuspended in PBS. *S*. Tm was fixed with PBS/2% PFA for 20 min, washed with PBS/1% bovine serum albumin (Cat# P06-1391100; PAN-Biotech) before staining with human anti-*S*. The Tm O12 antibody (hSTA5; kind gift from Antonio Lanzavecchia, Institute for Research in Biomedicine, Bellinzona, Switzerland) (1:200) was combined with goat anti-human-IgG AF647 (Jackson; #109-605-098) (1:200). The samples were incubated with the primary antibody for 30 min, followed by washing with PBS/1% bovine serum albumin and another incubation with the secondary antibody for 30 min. The stained bacteria were washed in PBS before acquisition with a Cytoflex flow cytometer (Beckman Coulter) using CytExpert software v.2.5. The gating strategy is provided in Figure S5A.

### Statistical analysis

Sample sizes (number of mice per group) were determined based on prior experimental experience, established practices for mouse models of *Salmonella* infections, and institutional animal welfare guidelines to minimize the number of animals used. No formal sample size calculation was performed, and therefore, no primary outcome measure was predefined for sample size determination.

To assess statistical significance, nonparametric statistical testing (two-tailed Mann‒Whitney *U* test) for mouse experiments was performed with GraphPad Prism 8 for Windows. Metabolite abundances were compared between the streptomycin- and ampicillin-pretreated groups using Mann–Whitney U tests followed by Benjamini–Hochberg correction for multiple testing, with an FDR threshold of 0.05. Undetected values were imputed as half of the lowest detected value for the respective metabolite. Because non-parametric statistical tests were used throughout, no formal assessment of data normality was performed. *P*-values: p ≥ 0.05 not significant (ns), *p* < 0.05(*), *p* < 0.01 (**), and *p* < 0.001 (***).

## Supplementary Material

Supplementary Material S1_Table_Bacterial_strains.xlsx

Supplementary Material StrepAmp_MS_FigS5.tif

Supplementary Material StrepAmp_MS_FigS4.tif

Supplementary Material StrepAmp_MS_FigS3.tif

Supplementary Material StrepAmp_MS_FigS2.tif

Supplementary Material StrepAmp_MS_FigS1.tif

Supplementary Material S5_Table_Calibrants_data.xlsx

Supplementary Material S4_Table_Results_of_weighted_linear_least_square_fitting_.xlsx

Supplementary Material S3_Table_MS_measurement_native_compounds.xlsx

Supplementary Material S2_Table_MS_measurement_organic_acid_and_sugar_hydrazones.xlsx

Supplementary Material Supplementary_captions.docx

## Data Availability

The sequencing data for this study are openly available in the European Nucleotide Archive (ENA) at https://www.ebi.ac.uk/ena/browser/view/PRJEB106337, reference number PRJEB106337. All other relevant data are within the paper and its Supporting Information files.
