# Supplementary material for: Antibiotic pretreatment with ampicillin or streptomycin differentially shapes Salmonella infection kinetics and associated intestinal inflammation in mice
Source: Gut Microbes. 2026 Sep 3;18(1):2725469. doi: 10.1080/19490976.2026.2725469 (PMC13556942; doi:10.1080/19490976.2026.2725469)
Supplement: Supplementary file 1 [file KGMI_A_2725469_SM1044.docx]

## Supporting figure legends

**S1 Figure. (WT C57BL/6 and 129S6/SvEvTac mice)**

(A-C) 4 d *S*. Tm^SPI2^ infection of SPF wild-type mice (C57BL/6) pretreated with either streptomycin (green circles) or ampicillin (red squares). (A) Experimental setup. (B) *S*. Tm^SPI2^ pathogen loads in feces at 1 d p.i. and 4 d p.i. (C) Quantification of inflammation by LCN2 levels of feces. The red dotted line indicates 200 ng/g feces, the threshold used to define inflammation. (D-F) 1d *S*. Tm^SPI2^ infection of SPF wild-type mice (129S6/SvEvTac) pretreated with either streptomycin (green circles) or ampicillin (red squares). (D) Experimental setup. (E) *S*. Tm^SPI2^ pathogen loads in feces at 1 d p.i. (F) Quantification of inflammation by LCN2 levels of feces. The red dotted line indicates 200 ng/g feces, the threshold used to define inflammation. In B, C, E, F, each data point represents one mouse (B, C: streptomycin n = 13, ampicillin n = 6; E, F: n = 6 per group, from ≥2 independent experiments). Line at median. The dotted line represents the detection limit. Mann-Whitney U test (ns–not significant, *P < 0.05, **P < 0.01).

**S2 Figure. (Supplementary figure to Fig. 2)**

Validation of successful microbial transfer by co-housing. (A) qPCR analysis of total fecal 16S rRNA gene levels using universal 515F/806R primers. Data show inverted raw Ct values from baseline GF controls, and from WT (recipient) and *GsdmD^-/-^* (donor) mice after >4 weeks of co-housing. DNA extracts were volumetrically standardized relative to starting fecal mass (0.1 g feces/mL). Samples with no amplification (GF) are indicated at Ct = 40. Each data point represents one mouse (GF control n = 3, WT (recipient) n = 6, *GsdmD^-/-^* (donor) n = 4). Line at median. The dotted line represents the limit of detection (Ct = 40). Mann-Whitney U test (ns–not significant).

**S3 Figure. (Supplementary figure to Fig. 3)**

(A-C) 1 d *S*. Tm^SPI2^ infection of GF WT mice (C57BL/6) pretreated with either streptomycin (green circles) or ampicillin (red squares). (A) Experimental set up. (B) *S*. Tm^SPI2^ pathogen loads in feces (C) Quantification of inflammation by LCN2 levels of feces. The red dotted line indicates 200 ng/g feces, the threshold used to define inflammation. In B, C, each data point represents one mouse (n = 4 per group). Line at median. The dotted line represents the detection limit. Mann-Whitney U test (ns–not significant). (D-E) *GsdmD^-/-^* mice were pretreated with either streptomycin (green circles) or ampicillin (red squares) and their gut microbiota composition was analyzed prior to treatment (untreated; -1 d p.i.) and one day post treatment (ABX pretreated; 0 d p.i.) by 16S rRNA gene sequencing. (D) Estimated absolute abundance of major bacterial phyla in the cecum content (belongs to Fig. 3D). Microbiota density was quantified by microscopy on 10 µm-thick tissue sections (Fig. 3A), and community composition was determined independently by 16S rRNA gene sequencing (Fig. 3D). As measurements were obtained from different mice within each group, the group median microbiota density was used to approximate total bacterial load. Relative abundances for each mouse were multiplied by the corresponding total bacterial load to estimate absolute abundances, expressed as estimated absolute abundance per mL cecum content. Note that these values represent an estimated absolute abundance and do not correct for variations in taxon-specific *rrn* copy numbers. (E) Relative abundances of ASVs at the order level based on 16S rRNA sequencing. The 13 most abundant orders are shown, with the rest of the community shown as “Others”. The x axis indicates the individual mice. In D, each data point represents one mouse (untreated n = 8, streptomycin n = 4, ampicillin n = 4). Data of D, E correspond to the same cohort of mice as in Fig. 3B-D. Line at median.

**S4 Figure. (Supplementary figure to Fig. 4)**

*GsdmD^-/-^* mice (C57BL/6 background) were pretreated with either streptomycin (green circles) or ampicillin (red squares) and the gut microenvironment was analysed one day post treatment. Untreated mice (empty triangles) were added as a control. (A) LC-MS-based absolute quantification of amino acids. (B) LC-MS-based absolute quantification of selected organic acids. Succinate concentrations were estimated from HILIC measurements using a 30 µM internal succinate standard, with absolute values calculated from analyte-to–internal standard peak area ratios assuming A₀/A_is_ = c₀/c_is_. ND = not detected; n indicates the number of samples below the limit of detection. Each data point represents one mouse (untreated n = 4, streptomycin n = 5, ampicillin n = 5). Data correspond to the same cohort of mice as in Fig. 3A and 4. For statistical testing and calculation of median fold changes, undetected metabolites were imputed with a value corresponding to half of the lowest detected value for the respective metabolite. Statistical comparisons between streptomycin- and ampicillin-pretreated groups were performed using Mann–Whitney U tests followed by Benjamini–Hochberg procedure for multiple testing. No metabolites remained statistically significant after FDR correction.

**S5 Figure. (Supplementary figure to Fig. 5)**

(A) Flow cytometry gating strategy using primary antibody human anti-*S*. Tm O12 and secondary antibody goat anti-human AF647 corresponding to Fig. 5B, C. Including a control where the primary antibody was not added (O12 FMO) and a control *S*. Tm^SPI2^ strain that does not express GFP (GFP FMO) (B-C) 4d *S*. Tm^∆^*^invG^*^∆^*^ssaV^* infection of *GsdmD^-/-^* mice pretreated with either Streptomycin (green circles) or Ampicillin (red squares). (B) *S*. Tm^∆^*^invG^*^∆^*^ssaV^* pathogen loads in feces. (C) Quantification of inflammation by LCN2 levels of feces. The red dotted line indicates 200 ng/g feces, the threshold used to define inflammation. In B, C each data point represents one mouse (n = 5 per group from ≥2 independent experiments). Line at median. The dotted line represents the detection limit. Mann-Whitney U test (ns–not significant, *P < 0.05).
